# Machine Learning Approaches for Automated Lesion Detection in Microwave Breast Imaging Clinical Data

**DOI:** 10.1038/s41598-019-46974-3

**Published:** 2019-07-19

**Authors:** Soumya Prakash Rana, Maitreyee Dey, Gianluigi Tiberi, Lorenzo Sani, Alessandro Vispa, Giovanni Raspa, Michele Duranti, Mohammad Ghavami, Sandra Dudley

**Affiliations:** 1 Division of Electrical and Electronic Engineering, School of Engineering, London South Bank University, London, United Kingdom; 2 UBT Srl, Spin Off of the University of Perugia, Perugia, Italy; 3 Department of Diagnostic Imaging, Perugia Hospital, Perugia, Italy

**Keywords:** Breast cancer, Cancer imaging, Biomedical engineering

## Abstract

Breast lesion detection employing state of the art microwave systems provide a safe, non-ionizing technique that can differentiate healthy and non-healthy tissues by exploiting their dielectric properties. In this paper, a microwave apparatus for breast lesion detection is used to accumulate clinical data from subjects undergoing breast examinations at the Department of Diagnostic Imaging, Perugia Hospital, Perugia, Italy. This paper presents the first ever clinical demonstration and comparison of a microwave ultra-wideband (UWB) device augmented by machine learning with subjects who are simultaneously undergoing conventional breast examinations. Non-ionizing microwave signals are transmitted through the breast tissue and the scattering parameters (S-parameter) are received via a dedicated moving transmitting and receiving antenna set-up. The output of a parallel radiologist study for the same subjects, performed using conventional techniques, is taken to pre-process microwave data and create suitable data for the machine intelligence system. These data are used to train and investigate several suitable supervised machine learning algorithms nearest neighbour (NN), multi-layer perceptron (MLP) neural network, and support vector machine (SVM) to create an intelligent classification system towards supporting clinicians to recognise breasts with lesions. The results are rigorously analysed, validated through statistical measurements, and found the quadratic kernel of SVM can classify the breast data with 98% accuracy.

## Introduction

Breast cancer is the most common cancer to affect women worldwide and the second most common cancer overall^1^, with nearly 1.7 million new cases diagnosed annually^2^. Mammography, the gold screening standard, is not suggested for screening women under 50 years old due to ionizing radiation exposure concerns. This means that 40% of all women in the EU (age 25–49 years old), representing 20% of breast cancer cases in Europe, cannot avail of the the most conventional breast cancer screening modality^3^. Furthermore, X-ray mammography cannot be undergone frequently, i.e. no more than once every 2 years in the EU, and it is prohibited for obvious reasons during pregnancy^4^. Breast cancer risk increases with further exposure to ionizing radiation from repeated mammography examinations^5^. Women who have undergone such tests also state that the exam is painful, particularly during their premenstrual period, or when the test is performed on women with smaller breasts^6^. Lastly, conventional mammography has been shown to miss approximately 15% of cancer (false negative)^7,8^. Bearing in mind these limitations, new imaging approaches must be considered. Hitherto, microwave imaging has gained increased attention for its potential in breast cancer detection scenarios, fortified by the measurable variations in the dielectric properties of malignant and normal tissues at the microwave frequency ranges used. Explicitly, the work presented by Li, Xu, *et al*.^9^ and Bond, Essex J., *et al*.^10^, demonstrated that a substantial contrast between malignant and healthy breast tissue is present; this contrast was demonstrated to be up to a factor of five in conductivity and permittivity. More recent works propose that this contrast is only between malignant and fatty breast tissues, with a lower contrast (lower than 10% in dielectric properties) is found between healthy fibro glandular and malignant tissues^11–13^. Moreover, Lazebnik, Mariya, *et al*., demonstrated that the dielectric properties of benign lesions are similar to the properties of fibro glandular tissues by^13^. Current microwave breast imaging research can be considered in two categories; microwave tomography and ultra-wideband (UWB) radar techniques^14^. A small number of prototypes are at clinical trial stages, including developments by Dartmouth College^15^ and the University of Bristol^16^. Specifically^15^, employs microwave tomography and employs antennas with a matching liquid, while^16^ employs an UWB radar approach and uses an array of 60 antennas with a matching liquid.

A UWB microwave prototype (Mammowave UBT etc) has been constructed, tested and validated previously^17^. The system operates in air employing two antennas and displays maps of dielectric property changes in tissues. Artefact removal (a matching liquid is not used here) is performed through appropriate mathematical procedures^18,19^. A Huygens Principle (HP) approach is used to capture differences in dielectric properties and discriminates tissues and tissue condition. Test on phantoms have shown a resolution of 1 mm^18^, while a sensitivity of 90% has been achieved in the ongoing clinical trial^19^.

Recently, machine learning based approaches for breast lesion detection have enjoyed increased attention^20–22^. Machine learning (ML) can be explicitly used to make decisions based on learned patterns (available datasets) and can automatically create an analytical model for future predictions without direct human intervention. Various methods such as nearest neighbor, neural networks, naive bayes, decision trees, conventional ML algorithms, and some hybrid approaches have been used for classification purpose. Also, deep learning (DL) based methods for tumor classification has been investigated. However, limited to breast lesion microwave imaging, ML and DL for breast lesion detection have been applied hitherto only to microwave datasets obtained through numerical simulations or measurements in phantoms^23–27^ and nor ever before to clinical data.

The authors present the first ever work on clinically trialed UWB data augmented by ML for automated safe breast lesion detection. The clinical trial UWB data have been collected at Perugia Hospital, Italy, using the microwave apparatus named “MammoWave”, a non-ionizing and X-ray free mammogram invented by UBT Srl, Italy. In this research, we have investigated the prospect of employing ML algorithms for computer-aided breast lesion detection to support clinicians, by reducing overhead and increasing the speed in decision making between healthy and non-healthy lesion patterns from the clinically collected data through the current microwave apparatus. Various ML algorithms have been applied in the field of pattern identification and future prediction. Among them, three popular methods, *k*-nearest neighbor (*k*NN), multi-layer perceptron (MLP) neural network, and support vector machine (SVM) are explored here to analyze the acquired labeled MammoWave data thoroughly. These experiments have been performed to fit the labeled training data with the optimal model parameters for predicting the presence of a lesion. The *k*NN uses a distance-based decision function for classifying lesions and MLP employs a nonlinear activation function to distinguish lesions. The obtained accuracy from these two classifiers is less than 60%, thus more suitable algorithms must be investigated to compare and establish the proposed work. Support vector machine has been investigated using a linear and quadratic kernel, which is faster and has achieved optimal prediction outcomes for lesion classification. These kernels are making SVM a powerful tool that can perform both linear and non-linear classification by mapping the inputs to a high dimensional feature space and separates the categories by a gap that is as wide as possible. The ML outcomes are evaluated through the results obtained from the radiologist’s report of the Perugia Hospital. These ML outcomes also have been validated through established statistical measures. Preliminary results show the proposed method produces minimal false-positives and false-negatives compared to other state-of-art methods and develop a viable anonymize method for mass screening breast lesion detection in future.

## Proposed Methodology

**Figure 1:**
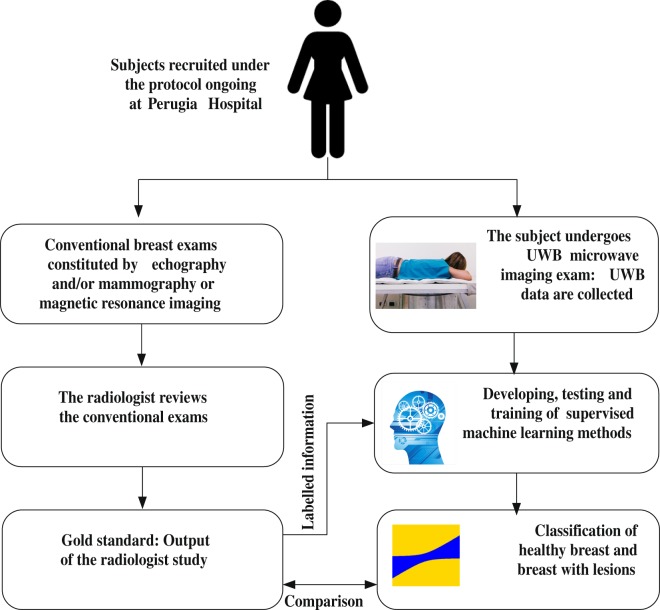
Flow diagram of the proposed work.

A “pipeline” schematic of the proposed work has been shown in Fig. 1. At first a subject undergoing conventional screening is asked to also undertake a parallel UWB imaging examination. In this case the conventional methods offered were echography, mammography, magnetic resonance imaging. The radiologist in charge reviewed the conventional imaging data as usual to make a decision regarding the screening outcome. The radiologist decisions have been considered as a gold standard identity of each breast type investigated. Then, these gold standard labels of the breasts have been employed to train the supervised machine learning algorithms to identify breast lesions automatically via the UWB imaging system. The outcomes of breast lesion detection from radiologists and ML have been compared to ensure system performance. The details of intermediate stages have been described in the following sections.

### Apparatus description and set-up

All of the UWB imaging data used in this paper were collected on subjects using the microwave prototype (MammoWave, UBT Srl, Italy) located at the Department of Diagnostic Imaging, Perugia Hospital, Perugia, Italy. All of the data was anonymized. The functioning principle of the MammoWave system is based on the dielectric property difference between normal tissue and tissue with lesions at microwave frequencies, i.e. the different behaviors that tissues display when irradiated by microwave signals.

**Figure 2:**
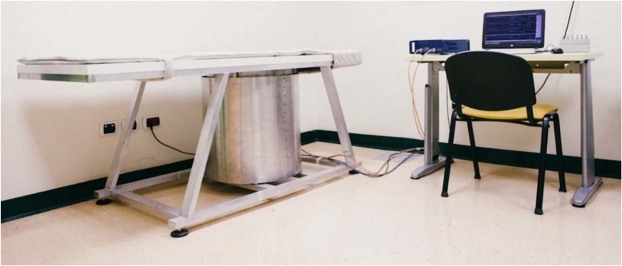
The UBT Microwave ‘MammoWave’ apparatus.

The hardware of the microwave prototype is composed of an aluminum cylindrical hub and shown in Fig. 2. The cylindrical hub represents a shield from external interferences and as a bearing structure for the entire device. On top of the cylindrical hub lies the examination bed. The bed incorporates a plexiglass cup aimed at containing the breast of the patient (facing down), with no pressure added to the breast tissue. Several sizes of this cup are available to accommodate the examination of different breast sizes.

**Figure 3:**
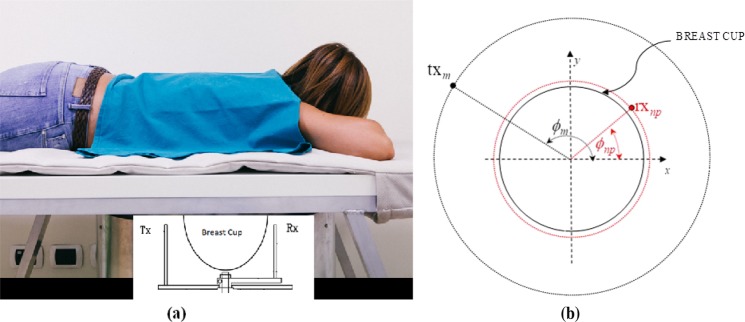
(**a**) Pictorial view of the system, where the transmit (*T*_*X*_) and receive (*R*_*X*_) antennas are placed inside the hub but external to the cup. (**b**) *T*_*X*_ (black dot) and *R*_*X*_ (red dot) can be moved around the azimuth, i.e. horizontal plane, on two circumferences having radius *a*_1_ and *a*_0_, respectively^44^.

The transmit and receive (*T*_*X*_ and *R*_*X*_ respectively) antennas are positioned inside the hub and external to the cup, as shown in Fig. 3. They can rotate around the azimuth, to irradiate the breast (through *T*_*X*_) and receive signals scattered by the breast itself (through *R*_*X*_). *T*_*X*_ and *R*_*X*_ have a distance from the center *a*_1_ = 20 cm and *a*_0_ = 7 cm, respectively. Both *T*_*X*_ and *R*_*X*_ are linearly polarized, operate in the 1–9 GHz frequency band and are connected to a vector network analyzer - VNA (Copper Mountain, Indianapolis, IN). Specifically, the received signals are the complex S21 data from the VNA. In particular, *R*_*X*_ can be rotated to measure the received signal at the points $r{x}_{np}\equiv ({a}_{0},{\varphi }_{np})$, displaced along a circular surface having radius *a*_0_, as shown in Fig. 3.

1 $${E}_{np,t{x}_{m}}({a}_{0},{\varphi }_{np};t{x}_{m};f)=S{21}_{np,t{x}_{m}}({a}_{0},{\varphi }_{np};t{x}_{m};f)$$

with $np=1,\ldots ,{N}_{PT}$. The device uses M positions of the transmitting antennas, i.e. *T*_*X*_ can be rotated to transmit the signal from the points $t{x}_{m}\equiv ({a}_{1},{\varphi }_{m})$, with *m* = 1, 2, …, M. Also, the device uses number of frequency (NF) samples in the band $B=[{f}_{{\min }}\div{f}_{{\max }}]$. The received signals are then processed through HP to calculate the field inside the cylinder; this field is then used to generate an image, which is a homogeneity map of dielectric properties^19^. Here, instead of using the received signals to generate images through the HP based algorithm, we employ ML methods on the raw signals to analyze and understand the difference between signals scattered from normal breast and breast with lesion to make decision about breast condition. The experiment was performed using Matlab R2017a tool in an Intel^*R*^ Core*™* i7 processor@ 3.60 GHz based Windows 7 Enterprise 64-bit operating system and it has 7856 MB NVIDIA Graphics Processing Unit (GPU).

### *In-vivo* acquisition

Continuous clinical recruitment of volunteers is underway at Perugia Hospital, Italy. The clinical validation for the first forty-five volunteers has been approved by the Ethical Committee of Regione Umbria, Italy, (N. 6845/15/AV/DM of 14/10/2015). More recently, another partner hospital, Foligno Hospital, also in Italy, has been approved by the Ethical Committee of Regione Umbria, Italy, to join the clinical validation trials. This will extend the number of subjects to one hundred and fifty (N. 10352/17/NCAV of 16/03/2017). This trial is a feasibility study of the method shown in Figs 2 and 3 employing microwave imaging to detect breast lesions, with the intention of gauging the potential of the proposed system for medical screening and localization of breast lesions. The list of a first pilot set of 18 subjects, who have been recruited under the aforementioned protocol and used for this study, is presented in Table 1. A previous table with 16 subjects was presented in^28^.

**Table 1:** Subject lists; details and related radiologist review.

Subject index and breast (left/right)	Year of birth	Breast type	Diagnostic test	Output of the radiologist study
R	1983	ACR4	ecography	Healty
L	1983	ACR4	ecography	Healty
R	1936	ACR2	mammography	carcinoma papillary
R	1960	N/A	magnetic resonance	carcinoma infiltrating grade 2
R	1987	ACR4	ecography	Healty
L	1987	ACR4	ecography	Healty
L	1987	N/A	ecography	benign fibroadenoma
L	1975	ACR2	ecography + mammography	benign fibroadenoma
R	1980	ACR3	ecography + mammography	benign microcalcifications
R	1929	ACR4	ecography + mammography	carcinoma
R	1963	ACR3	mammography	Healthy
L	1963	ACR3	mammography	benign fibroadenoma
L	1964	ACR4	mammography	Healthy
R	1964	ACR4	mammography	Healthy
R	1946	ACR2	mammography	Healthy
R	1966	ACR3	mammography	carcinoma (4 cm), b5
L	1971	ACR3	mammography	carcinoma
L	1996	N/A	ecography	benign fibroadenoma
R	1969	ACR4	mammography	microcalcifications
R	1948	ACR2	mammography	Healthy
R	1971	ACR4	mammography	Healthy
L	1971	ACR4	mammography	Healthy
R	1983	ACR3	mammography	Healthy

Overall shows 12 healthy and 11 non-healthy tissues. Included in the non-healthy is one post-surgical breast with seroma.

All volunteers provided informed consent with five subjects undergoing the proposed microwave imaging for both breasts. Thirteen subjects underwent the prototype imaging as shown in Fig. 3 for a single breast. This research was conducted adhering to the ethical standards of the institutional and/or national research committee incorporating the 1964 Helsinki declaration and its later amendments or analogous ethical standards. The study was performed in harmony with the Code of Ethics of the World Medical Association for experiments involving humans.

A conventional exam and full radiologist study was performed for each subject; echography and/or mammography or (limited to one case) magnetic resonance imaging were the conventional exams performed in these cases. The MyLab 70 xvg Ultrasound Scanner (Esaote, Italy) was the echographic method used; A Selenia LORAD Mammography System (Hologic, Marlborough, USA) was employed for mammography examinations; and a 3 T scanner (Siemens Healthcare, Germany) was used for magnetic resonance imaging. The radiologists diagnosis is listed in Table 1 which presents the outcomes from the radiologists report along with the subject’s breast condition details. Where available, the breast type has been classified according to density, as defined by the American College of Radiology (ACR) scale ranging from ACR1 (extremely fatty breast) to ACR4 (extremely heterogeneous fibroglandular breast)^29^. If present, the inclusion type was classified according to defined standards^30–32^.

Following a volunteer’s agreement to participate, the clinical study coordinator supports each volunteer to place their breast correctly into the system cup. This cup is integrated into the prototype bed for improved comfort and patient stability. Exam data is processed via a computer interface and is observed by a system operator located in the room. Overall, the exam and data collection phase require approximately five minutes per breast. The transmit and receive antennas (*T*_*X*_ and *R*_*X*_ are positioned on the azimuth plane at the same height which crosses the center of the breast of the subject being examined (after ensuring that the antennas half power beam angle include the breast). *M* = 15 transmitting positions are employed, divided in 5 groups centered at 0°, 72°, 144°, 216°, and 288° on the azimuth plane; each group has 3 transmitting positions displaced from each other by 4.5°. For each transmitting position, four receiving positions, at 90° from each other are employed. The S21 was acquired at *NF* = 1601 frequencies from 1 to 9 GHz in Δ*f* = 5 MHz increments for each *T*_*X*_ and *R*_*X*_ position. The received signals are processed as follows:

1. Let us consider the first transmitting antennas group and the antennas in the group are numbered as *tx*_1_, *tx*_2_, *tx*_3_; the first transmitting group is assumed to be centered at $\varphi =0^\circ$. For each transmitting antenna of the group, we consider the signals received at ${N}_{PT}=4$ points displaced at $\varphi =45^\circ ,135^\circ ,225^\circ ,315^\circ$ with respect to the corresponding transmitting antenna.
2. To remove skin artefacts, we generate the following signals:
  2 $${E}_{1-2}({a}_{0},{\varphi }_{np};t{x}_{1-2};f)={E}_{np,t{x}_{m}}({a}_{0},{\varphi }_{np};t{x}_{1};f)-{E}_{np,t{x}_{m}}({a}_{0},{\varphi }_{np};t{x}_{2};f)$$
  3 $${E}_{2-3}({a}_{0},{\varphi }_{np};t{x}_{2-3};f)={E}_{np,t{x}_{m}}({a}_{0},{\varphi }_{np};t{x}_{2};f)-{E}_{np,t{x}_{m}}({a}_{0},{\varphi }_{np};t{x}_{3};f)$$
  where, $${\varphi }_{np}=45^\circ ,135^\circ ,225^\circ ,315^\circ $$
3. The procedure is repeated for the other four transmitting groups. It follows that, for each microwave exam, we have 40 signals in the frequency domain.

### Supervised Machine Learning

The data gathered from the microwave examination are considered for classification purpose and/or to predict future lesion detection instances. This is the first investigation to use microwave clinical data from this apparatus undergoing machine learning classification. Labeled information about the healthy and non-healthy breast pattern have been gathered from the output of the radiologist study. Specifically, a healthy breast is a breast with no lesion, while a non-healthy breast is a breast containing a lesion which may be benign or malignant. The accumulated clinical data have high variance in Euclidean space, so both linear and non-linear classifiers are employed to optimised performance for identifying healthy and non-healthy subjects. There are several ML algorithms present for classification tasks in the literature, some of them being very problem specific while others aim to be more general requiring an investigation approach to be fitted to available data. The selection of appropriate ML methods is quite intuitive and the data distribution in the plane needs to be initially observed. It has been found that the gathered microwave data are non-linearly distributed in the plane. Thus, the leading supervised and non-linear classifiers, KNNs, MLPs, SVMs have been examined, where cross validation techniques, augmented by random sub-sampling methods are employed to asses the performance, through statistical metrics, and discover the most appropriate classification model. Initial results from non-linear classifiers such as, KNNs and MLPs were unsatisfactory. The radial basis function (RBF) could be an option, but it performs well where the data are in loop or spherical shape and circular decision boundary can differentiate the groups. Though, the data are non-linear they are not spherically distributed, hence the SVM has been implemented with a linear kernel function because, although the data distribution appears non-linearly separable in the 2D plane, there is still a possibility to classify most lesion instances accurately by linear decision boundary while the data are being projected into hyperplane i.e., impossible to visualise. The linear kernel of SVM also failed to achieve satisfied performance, and SVM has been experimented with quadratic kernel function which outperformed other classification techniques.

### Cross validation and performance evaluation

A cross validation technique has been used to assess, enhance predictive outcomes, and select models for developed ML prototypes. This has been done by repeated random sub-sampling of the data, which is also known as Monte Carlo cross-validation^33^. The dataset has been randomly partitioned to select the training and validation dataset, where training and validation sets have been used to train and evaluate performance of a selected ML model. The ratio of training and testing data has been specified during each round e.g., training has been started with 10% randomly selected data when rest of the 90% data have been considered as validation/testing data. The amount of training data has been increased by 10% while amount of validation dataset decreased by 10% in each round and this process has been repeated till the model has not overfitted. Each model has been run 25 rounds to select the appropriate ratio of training and testing and found 40% of training and 60% of testing data is necessary to prevent the ML algorithms from overfitting. The results (statistical metrics) have been aggregated and averaged over all the rounds. A number of statistical metrics^34^, accuracy, true positive rate (TPR) or sensitivity, true negative rate (TNR) or specificity, positive predictive value (PPV), and negative predictive value (NPV) have been used to investigate the classification performance of the classifiers. A receiver operating characteristic (ROC) curve has been generated for each ML model with the validation dataset to illustrate the diagnostic ability and stability of the classification system with different discrimination threshold. Subsequently, Matthews Correlation Coefficient (MCC)^35^ and Youden’s index^36^ have been implemented to investigate the classification outcomes, where, MCC and Youden’s index estimate quality of classification and probability of the informed decision respectively. The outcomes and it’s analysis have been described in next section.

## Results Analysis

**Figure 4:**
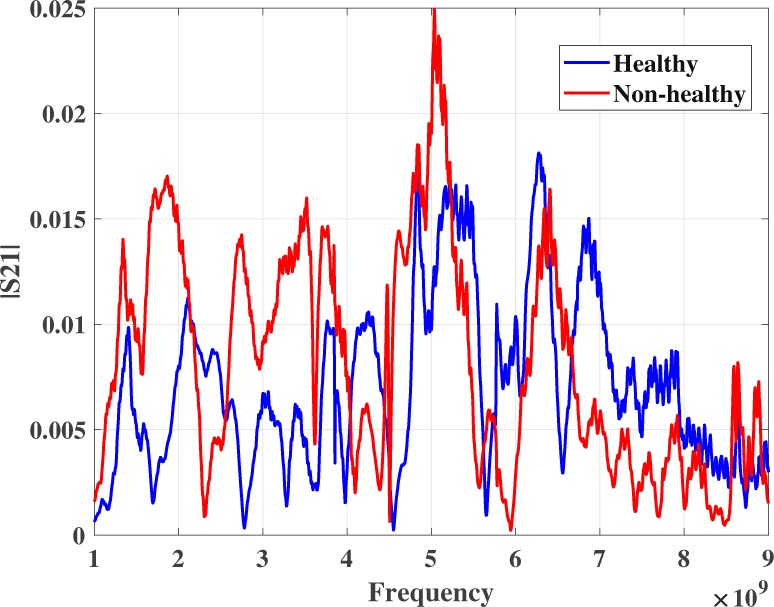
Example signals of healthy and non-healthy patterns in 45°.

According to the radiologist’s review, 12 healthy breasts and 11 non-healthy breasts, i.e. breasts with lesions, underwent the microwave exam of the 18 subjects. As described in the previous section, each microwave exam leads to 40 different patterns in the frequency domain. As an example, Fig. 4 below shows ${E}_{1-2}({a}_{0},{\varphi }_{np};t{x}_{1-2};f)$ for ${\varphi }_{np}=45^\circ$ for one healthy and one non-healthy breast.

### *k*-nearest neighbor classifier

Initially, the investigation began by employing the *k*-NN classifier^37^. The classifier is particularly simple, measuring the proximity of features in the hyperspace without any assumption of the underlying data distribution to predict a category, making it flexible for decision making. Two effective distance metrics, Euclidean and Mahalanobis perform well with *k*-NN, but Mahalanobis distance requires the inversion of covariance matrix which could increase the computational overhead. Therefore, the Euclidean distance is considered here to measure the distance of a feature vector from its nearest neighbor. The *k* is chosen as odd for this two-class problem that one pattern could not predict under the same class label by the classifier. Table 2 displays the classification outcomes, where *k* is varied from 1 to 5 and 10%, 20%, 30%, and 40% data are randomly selected for the training phase. The results show that the algorithm exhibited good performance with increasing training data volume as expected. Here, *k* = 1, produces the optimal result among other NNs with 40% of training data volume. It attained the testing accuracy 0.608 (≡60.8%). TPR or sensitivity measures the ability of the algorithm to identify the non-healthy subjects, which is 0.541(≡54.1%) in the case of *k* = 1. It could correctly identify the subjects with lesions with a rate of 0.667 (≡66.7%). PPV and NPV are influenced by the prevalence of having a lesion in the breast that is being tested. In case of *k* = 1, PPV and NPV the probability that the subjects with positive lesion identification truly have the lesion and negative identification of lesions truly do not have the lesion. The 1NN produces fewer false predictions close to the decision boundary bringing improved accuracy over 3NN and 5NN; also, truly positive prediction for having lesion and vice versa. Additionally, the MCC measurements over prediction results are also not cogent for 1NN, 3NN, and 5NN. The average MCC is approximately 0.206 in case of 1NN and decreases towards 0 with the increment of *k*. This trend states that the addition of random predictions with greater number of NN. The average proportions obtained from Youden’s index are also very low, 0.206, 0.130, and 0.086 for 1NN, 3NN, and 5NN respectively further indicating the probability to predict those lesions is random and unreliable. This index works along with the ROC curve, the outcomes have been correlated at the discussion of ROC analysis. Therefore, the overall performance of 1NN is better than the other NNs because of data compactness, where one nearest neighbor results in a good prediction if a greater number of neighbors have been chosen, the misclassification increases.

**Table 2:** Results obtained from nearest neighbor algorithm.

NNs	% of Training Data	Accuracy	Sensitivity	Specificity	PPV	NPV	MCC	Youden’s Index
1NN	10	0.555	0.567	0.545	0.521	0.590	0.203	0.202
	20	0.551	0.441	0.650	0.532	0.563	0.225	0.225
	30	0.586	0.580	0.592	0.551	0.620	0.186	0.186
	40	0.608	0.541	0.667	0.587	0.624	0.210	0.209
3NN	10	0.519	0.648	0.405	0.488	0.569	0.143	0.142
	20	0.528	0.448	0.600	0.505	0.544	0.125	0.124
	30	0.559	0.460	0.652	0.553	0.562	0.123	0.122
	40	0.568	0.520	0.609	0.531	0.598	0.134	0.132
5NN	10	0.532	0.434	0.619	0.499	0.555	0.084	0.082
	20	0.541	0.412	0.656	0.517	0.555	0.088	0.087
	30	0.549	0.577	0.524	0.513	0.588	0.092	0.089
	40	0.550	0.419	0.665	0.522	0.567	0.082	0.081

### Multilayer perceptron classifier

Two different multilayer perceptron^38,39^ algorithms are studied where, each algorithm is created with one hidden layer and the number of nodes in the hidden layer are decided using a ‘rule of thumb’ ($\sqrt{(number\,of\,inputs+number\,of\,outputs)}$ + (*a constant between* 1 *to* 10 *set by experimentally*)). The optimal size of the hidden layer is decided typically between the size of the input and output layers. The bias and weights are initialized randomly, the learning rate $\eta =0.1$ is varied up to 0.99. The output of the layers is determined by the hyperbolic tangent sigmoid transfer function. The Mean Square Error $({\rm{MSE}})=\frac{1}{N}\,{\sum }_{i=1}^{N}\,{({t}_{i}-{a}_{i})}^{2}$ is calculated for each output to back-propagate and update the weights, where *t* and *a* signify the targets and outputs, respectively.

First, the network is trained using the Levenberg-Marquardt (LM) algorithm which adaptively varies the parameter updates and performs better (because of the weight updation using a damping coefficient) than the simple gradient decent method that defines simple first order iterative optimization function and finds the local minimum, local maximum for parameter updating. The training stops when the maximum number (=1000) of epochs is reached, where one set of weight updating using backpropagation is considered as one epoch. Table 3 presents the results for both MLP using Levenberg-Marquardt *MLP*_*LM*_^40^ and Bayesian-Regularization (BR) backpropagation *MLP*_*BR*_^41^. In the case of *MLP*_*LM*_, the testing accuracy increases for up to 40% training data, but the increment rate is negligible, and reached 0.532 (≡53.2%), but results 0.076 (≡7.6%) sensitivity or TPR indicates the network can only identify 7.6% subjects correctly. However, it shows good performance in recognizing subjects without lesions (TNR = 0.936). Also, the probability of the prediction for identification of subjects having or not having lesions is slightly more than 50%. In the case of *MLP*_*BR*_, the overall performance is similar to *MLP*_*LM*_. The maximum testing accuracy reached 0.538 (≡53.8%) when 40% training data is used but results only 0.038 TPR demonstrates the inability to make predictions about lesions whereas 0.951 specificity or TNR shows a strong performance in predicting the absence of a lesion. Therefore, neither MLPs could predict the healthy breast pattern, but did make acceptable predictions for subjects with lesions. The probability of the prediction for identification of subject with or without lesions is between 0.511 to 0.542 for both *MLP*_*BR*_ and *MLP*_*LM*_. Additionally, the estimation of MCC and Youden’s statistic state insignificant power of MLPs to identify breast lesions. MCC of *MLP*_*LM*_ and *MLP*_*BR*_ are 0.019 and 0.082 respectively, implies the performance of MLPs are no better than random prediction with a large, unacceptable, misclassification rates. Subsequently, Youden’s statistics of *MLP*_*LM*_ and *MLP*_*BR*_ are 0.018 and 0.082 respectively. The zero tendency of the indices show the high proportion of false positives and false negatives. Misidentifications have been found here because the error surfaces are very complex for both of these networks and have stagnant into several local minima, producing unexpected outcomes for healthy and non-healthy patient identification.

**Table 3:** Results obtained from multilayer perceptron algorithm.

MLPs	% of Training Data	Accuracy	Sensitivity	Specificity	PPV	NPV	MCC	Youden’s Index
*MLP*_*LM*_	10	0.498	0.111	0.847	0.394	0.514	0.020	0.019
	20	0.530	0.052	0.962	0.551	0.529	0.010	0.009
	30	0.531	0.190	0.825	0.482	0.542	0.016	0.015
	40	0.532	0.076	0.936	0.511	0.534	0.031	0.030
*MLP*_*BR*_	10	0.532	0.035	0.953	0.428	0.532	0.081	0.081
	20	0.533	0.032	0.934	0.462	0.513	0.088	0.088
	30	0.538	0.037	0.957	0.414	0.527	0.082	0.082
	40	0.538	0.038	0.951	0.425	0.538	0.078	0.078

### Support vector machine classifier

Subsequently, the SVM is investigated with two different kernel functions to acquire the hyperplane that can separate healthy and non-healthy subjects. Table 4 shows the results for classification of the 2 subject types where, *SVM*_*L*_ and *SVM*_*Q*_ represents the SVMs using the linear and quadratic kernel functions^42,43^ for prediction. *SVM*_*L*_ uses the optimization method, $c={\sum }_{i}\,{w}_{i}k({s}_{i},x)+b$ where, subject pattern vector *x* is targeted to classify, *s*_*i*_ is the support vector, *w*_*i*_ is weight, and *b* is the bias. Here, the linear kernel function is *k*. The vector *x* is considered a member of the lesion free group when, $c\ge 0$ or lesion group otherwise. This creates a hyperplane that achieved better accuracy than the other classifiers used above. *SVM*_*L*_ is trained using 10% to 40% data and associated testing results are shown in the table. It produces the highest testing accuracy with 40% training data which is 0.620 (≡62.0%). It achieved TNR of 0.998 (≡99.8%) in that case, which indicates a good performance to identify the subjects with no lesions, but TPR (maximum 0.193 ≡ 19.3% among all cases of *SVM*_*L*_) shows a very weak performance in identifying subjects with lesions. Though the probability in identifying subjects who truly have lesions is better than the subjects who truly do not have lesions. The number of false negatives are continuously high, but false positives are low, which resulting an average MCC 0.319 for *SVM*_*L*_. Though, MCC is better than the other algorithms, it is still not powerful enough to reduces false negatives. The average Youden’s index is 0.180 which is close to the Youden’s index obtained from 1NN beacuse, *SVM*_*L*_ produced more false negatives and fewer false positives, whereas 1NN resulted in fewer false negatives and more false positives, thus the total number false predictions are high in both the cases.

**Table 4:** Results obtained from SVM using different kernel.

SVMs	% of Training Data	Accuracy	Sensitivity	Specificity	PPV	NPV	MCC	Youden’s Index
*SVM*_*L*_	10	0.620	0.191	0.997	0.981	0.584	0.319	0.182
	20	0.619	0.184	0.998	0.985	0.584	0.318	0.178
	30	0.616	0.194	0.996	0.977	0.579	0.324	0.180
	40	0.620	0.171	0.998	0.989	0.589	0.318	0.181
*SVM*_*Q*_	10	0.985	0.969	0.996	0.985	0.973	0.959	0.956
	20	0.984	0.967	0.997	0.983	0.972	0.963	0.960
	30	0.984	0.965	0.996	0.985	0.970	0.963	0.959
	40	0.989	0.977	0.997	0.985	0.981	0.955	0.951

**Figure 5:**
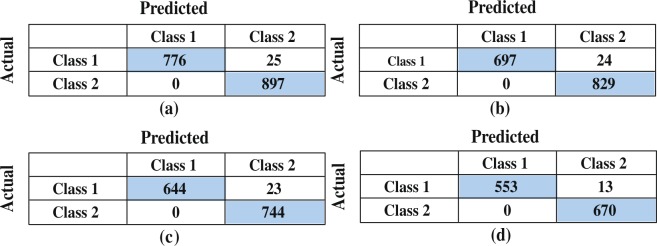
Confusion Matrices of *SVM*_*Q*_: (**a**) 10% training data, (**b**) 20% training data, (**c**) 30% training data, and (**d**) 40% training data are used.

Subsequently, *SVM*_*Q*_ have been employed to obtain an improved testing accuracy to differentiate subjects by minimising the gap between the two groups. The considered quadratic function is ${{\rm{\min }}}_{x}\frac{1}{2}{x}^{T}Hx+{c}^{T}x$, where $Ax\le b$, *c* is a real valued vector, *H* is real symmetric matrix, *A* is real matrix, b is a real vector, and the notation $Ax\le b$ means that every entry of the vector *A*_*x*_ is less than or equal to the corresponding entry of the vector *b*. The quadratic programming aims to find the vector *x* which could minimize that function. This function creates the best hyperplane to classify the subjects here. *SVM*_*Q*_ achieved 0.989 (≡98.9%) testing accuracy to identify the lesion affected and unaffected subjects. Correspondingly, TPR (is 0.9770 ≡ 97.70%) and TNR (is 0.997 ≡ 99.7%) both are high in this case, which indicates a good implementation of the hyperplane for separation which could correctly identify those subjects with and without lesions. Also, the high probabilities (PPV and NPV) support the results. In addition, MCC and Youden’s index both are high (i.e., 0.960 and 0.956 respectively) in this case. Figure 5 illustrates the outcomes more clearly, where no false positives, and few false negatives have been found in each run. This greatly influences the score of MCC and Youden’s index and proves the strong ability of the *SVM*_*Q*_ model to identify breast lesions.

Figure 5 shows the outcomes of *SVM*_*Q*_ more closely from the confusion matrices. The numeric values listed in the confusion matrix with a blue background demonstrates the correct classification of lesion affected and unaffected patterns. Very few misclassification occurred here (between 1.1% to 1.5%). Without lesion and with lesions are denoted by class-1 and class-2, respectively. It is shown that all the normal tissue subjects are classified correctly, few misclassifications are found with the training data variation (10% to 40%) in each case of *SVM*_*Q*_. The most important part of this lesion classification is to reduce the negative predictions (including false positive and false negative). It is found from the confusion matrices that the false prediction rate of lesion detection is zero in each case (all the patterns of class-2 are being predicted as class-2). False positive rate (FPR) is zero (from Fig. 5a–d) for all the cases of *SVM*_*Q*_, which implies all the non-lesion breast patterns are correctly classified but, few false negative rates (FNR) occurred, 0.027 (2.7% for 10% training data), 0.028 (2.8% for 20% training data), 0.029 (2.9% for 30% training data), and 0.019 (1.9% for 40% training data). Results here are far exceed other state-of-art methods reported to miss 15% of lesions. The effect of this classification and misclassification has been discussed earlier.

**Figure 6:**
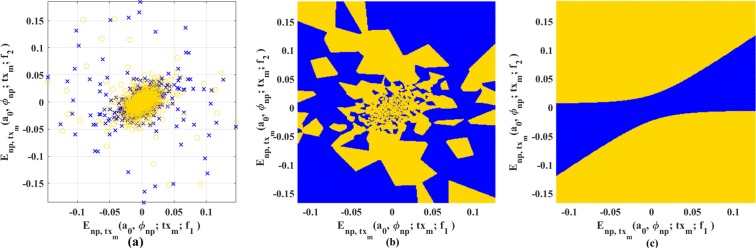
(**a**) Actual data distribution in two-dimensional plane, (**b**) decision boundary produced by 1NN classifier, (**c**) decision boundary produced by SVM with quadratic kernel.

As reported, *SVM*_*Q*_ has achieved the best performance among the classifiers investigated here in terms of accuracy, sensitivity, and specificity. It’s important here to visualize the data with corresponding hyperplanes formed by the ML algorithms. Figure 6 presents the data distribution along with the two optimal decision boundaries produced by 1NN and *SVM*_*Q*_. Figure 6a represents the breast with lesion and healthy breast data distribution in a two-dimensional feature space for visualization of the studied data pattern for the classification purpose. All the healthy breast patterns scatter the microwaves in a particular way whereas a breast lesion may occur in any position of the breast, which produces different scattering effect for the breasts with lesion. Hence, it has been found healthy data formed the cluster in the center and lesion data scattered around that group. Thus, these two groups of data could only be handled by the parabolic or quadratic curve, which has been made by *SVM*_*Q*_ here (decision boundary of Fig. 6c). In addition, Fig. 6b shows the decision boundary to correlate the misidentifications obtained by 1NN algorithm, which also have been illustrated in Table 4.

**Figure 7:**
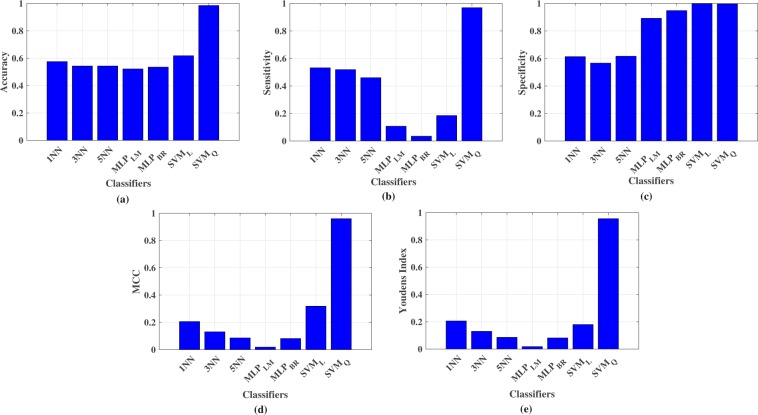
Comparison of performance metrics for all classifiers, (**a**) average accuracy, (**b**) average sensitivity, (**c**) average specificity, (**d**) average MCC, (**e**) Youden’s index.

Finally, the obtained classification results are compared to conclude the investigation. Figure 7 shows the visual comparison of average classification accuracy, sensitivity, specificity, MCC, and Youden’s index to make the contrast over performance, where *x* and *y*-axis represent different classifiers and accuracy, sensitivity, specificity, MCC, and Youden’s index respectively. It can be seen, the performance of *SVM*_*Q*_ is better than other classifiers attempted here in terms of average accuracy (98.55%), sensitivity (96.95%), specificity (99.65%), MCC (96%), and Youden’s statistic (95.6%) which illustrates robust ability to detect breast lesions from new microwave data.

**Figure 8:**
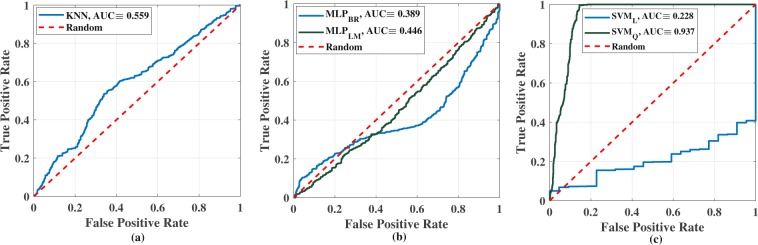
ROC curve analysis of the classifiers, (**a**) KNN, (**b**) MLP using Levenberg-Marquardt and Bayesian-Regularization backpropagation functions, (**c**) SVM using linear and quadratic kernels.

The ROC curve has also been plotted and shown in Fig. 8 to compare and analyse the diagnostic ability of all three classifiers, where the number of nearest neighbour, learning rate, and threshold for detection have been varied for NNs, MLPs, and SVMs respectively. The area under curve (AUC) has also been determined for each classifier. The *x* and *y*-axes of Fig. 8a–c represent false positive rate (FPR) or (1-specificity) and true positive rate (TPR) or sensitivity respectively. Though, the accuracy of 1NN is better than other classifiers, except *SVM*_*L*_ and *SVM*_*Q*_, the AUC of overall KNN (Fig. 8a) is only 0.599 indicating the large presence of false predictions. In the case of *MLP*_*BR*_ and *MLP*_*LM*_, AUCs are only 0.389 and 0.446 respectively. Both the MLPs produce vast amount of false predictions, as discussed earlier, and generate low AUC, and the tendency of performance is highly random in terms of all performance metrics. Though, the accuracy of *SVM*_*L*_ is approximately 0.619, it made a large number of false predictions and produced an AUC of 0.228 when the threshold had been varied. The highest AUC (≡0.937) is created by *SVM*_*Q*_ as this delivers the lowest number of false predictions among all tested and scored the highest performance metric for all the cases.

The parametric (i.e., MLP, and SVM) and non-parametric (i.e., KNN) both types of learning techniques have been used here to obtain optimal performance on the currently available dataset. The work has two main limitations, high dimensional feature space and availability of data (i.e., still ongoing). The dimension of the feature space has not been reduced in this study and considered as future scope. The KNN and SVM make the decision based on the similarity measure, whereas MLP depends upon features. The non-parametric KNN does not require any assumption of feature distribution to check similarity, but it requires a large amount of uncorrelated and independent training data in order to make good predictions, whereas the data used in this study are highly correlated and high dimensional in nature. Thus, the KNN has overfitted with 40% of training data, the overall performance is unsatisfactory and also deteriorated with the increment of *k*. Both the MLPs *MLP*_*BR*_ and *MLP*_*LM*_ are parametric in nature and make assumptions of feature space to minimise cost function and get optimised weights. Though, the function LM which directs *MLP*_*LM*_ is well known for optimising cost function but, choice of damping parameter played vital role for the study and the model stuck in local minima within 50 epochs which prevents the model to optimise weights and create good decision boundary. Thus, the damping parameter needs further tune to obtain good results. The Bayesian-Regularization (BR) is a well known optimisation technique that works well with MLP even if the data are high dimensional. But, the possible reason for incorrect predictions of *MLP*_*BR*_ is miss-specification of the model which indicates the function model does not suit BR for this problem. The SVM has been used by two different kernel functions, linear (*SVM*_*L*_) and quadratic (*SVM*_*Q*_). The methods rely on similarity, unlike the KNN model, SVMs is sensitive to the curse of dimensionality problem while the the features are not engineered to uncorrelated values. *SVM*_*L*_ is well known for its ability to separate non-linear data linearly in the higher feature space. Here, the dot product weight and features with the conventional bias have been employed to create a linear hyperplane and maximise the gap between support vectors and samples. Also, the estimation of bias is trivial in this case, thus, the maximisation of the linear kernel function created hard margins and increased errors as a result. On the other hand, *SVM*_*Q*_ is an extended version (2^*nd*^ order polynomial) of *SVM*_*L*_ which creates a soft margin in the feature space. The *SVM*_*Q*_ is focused on the minimisation problem. The kernel has two other variables, *c* and *x* which denote penalty constraint and the slack variable respectively. This is the advantage found using *SVM*_*Q*_, where *x* minimise error and *c* minimise the gap at the same time so that the non-linear boundary has been formed for one group (healthy or non-healthy) and rest of the samples fall in the other side of the boundary.

## Discussion

The frequency domain scattering parameters of subject breasts are obtained using the UWB microwave mammogram apparatus described above in an ongoing clinical trial. The same subject breasts included in this study have also undergone radiologist scrutiny obtained using conventional imaging methods (echography and/or mammography or (limited to one case) magnetic resonance imaging), which has been used as labeled information. The microwave mammogram apparatus clinical data are pre-processed and transformed for machine learning approaches. Various algorithms are trained and tested to differentiate lesion-containing and lesion-free breast tissues. Here, breasts with lesions may be benign or malignant. The experimental results show that the quadratic kernel of SVM has successfully created the hyperplane and maximizes the margins between the support vectors, resulting in a sensitivity for breast lesion classification equal to 97%. Such value outperforms the sensitivity given in^16^ and^17^, which are 74% and 90% respectively, where machine learning is not employed. The successful employment of machine learning on clinical data obtained using a microwave mammogram could help the radiologist in the diagnosis process. The integrated system with microwave non-ionizing imaging augmented by machine learning algorithms can be a step change in mass breast screening deployment. The system could be deployed across all female age groups, and during pregnancies, in more local settings, increasing the detection and hence survival rates of breast cancer sufferers.

This study aimed to differentiate between breast with or without lesions, but the type of lesion cannot yet be identified at this stage. The authors are currently gathering more clinical data to understand the category and property of lesions through the MammoWave device. Once more data have been gathered to make generalized decisions about lesions, this work will be extended further to identify the type of lesions automatically using ML. Although, the *SVM*_*Q*_ has worked very well in terms of all statistical performance metrics, the study is limited by some factors and those are considered for future work. The data used here have a high dimension and values are correlates, which placed the experimented ML methods in the curse of dimensionality problem. Thus, suitable dimensionality reduction techniques will be investigated in the next stage. The analysis of ROC curve shows that performance improvement for all experimented ML algorithms are possible. This study is an empirical study on ML application of automatic lesion detection to investigate suitable classifiers to categorise the data in hyperspace. The performance analysis directs focus on hyperplanes created by a quadratic function, thus the fine tuning of the parameters such as, slack variable and penalty parameter would be observed subsequently. Also, the parameter bias of other algorithms would be taken into account since the performance of current prototype may vary when the type of lesion will be identified with new datasets. Expert clinical input will be ensured in further research to meet clinical expectations in the assistance of breast lesion identification and classification. In addition, deep learning approaches will be investigated to provide higher sensitivity and specificity towards automation.
